# State of the medical physics profession in 2019

**DOI:** 10.1002/acm2.12513

**Published:** 2018-12-20

**Authors:** Michael Mills

How would you describe AAPM and medical physics culture? How would you evaluate our leadership, our governance processes, our ethics, management practices, and trust? What should it look like if we were really successful? One metric is to compare how we view our culture with historical benchmark elucidations of organizational success. Is there a way forward? I am encouraged that we have already seen many examples of the right way to organize in our history. I am especially impressed with the “Hewlett‐Packard Way.”

“The HP Way (c. 1992).”

We have trust and respect for individuals.

We approach each situation with the belief that people want to do a good job and will do so, given the proper tools and support. We attract highly capable, diverse, innovative people, and recognize their efforts and contributions to the company. HP people contribute enthusiastically and share in the success that they make possible.

We focus on a high level of achievement and contribution.

Our customers expect HP products and services to be of the highest quality and to provide lasting value. To achieve this, all HP people, especially managers, must be leaders who generate enthusiasm and respond with extra effort to meet customer needs. Techniques and management practices which are effective today may be outdated in the future. For us to remain at the forefront in all our activities, people should always be looking for new and better ways to do their work.

We conduct our business with uncompromising integrity.

We expect HP people to be open and honest in their dealings to earn the trust and loyalty of others. People at every level are expected to adhere to the highest standards of business ethics and must understand that anything less is unacceptable. As a practical matter, ethical conduct cannot be assured by written HP policies and codes; it must be an integral part of the organization, a deeply ingrained tradition that is passed from one generation of employees to another.

We achieve our common objectives through teamwork.

We recognize that it is only through effective cooperation within and among organizations that we can achieve our goals. Our commitment is to work as a worldwide team to fulfill the expectations of our customers, shareholders and others who depend upon us. The benefits and obligations of doing business are shared among all HP people.

We encourage flexibility and innovation.

We create an inclusive work environment which supports the diversity of our people and stimulates innovation. We strive for overall objectives which are clearly stated and agreed upon, and allow people flexibility in working toward goals in ways that they help determine are best for the organization. HP people should personally accept responsibility and be encouraged to upgrade their skills and capabilities through ongoing training and development. This is especially important in a technical business where the rate of progress is rapid and where people are expected to adapt to change.” (https://www.inflexion-point.com/Blog/bid/74097/5-Timeless-Principles-Revisiting-the-HP-Way). Replace “HP people” with medical physicists, and we have a good roadmap for the professional culture we want to build, continually.

When I began training in medical physics in 1974, I worked with one of the first treatment planning computers, the Artronix PC‐12. (https://en.wikipedia.org/wiki/Artronix). (https://www.researchgate.net/figure/The-Artronix-PC-12-treatment-planning-computer-rho-theta-transducer-A-tapedeck-B_fig 1_12217056). Calculation of a single dose distribution could take all night. Mantle field calculation was so slow, you could race it by hand to see if you could beat it. Still, that early treatment planning computer was a foretaste of the explosion of the information manipulation and management to come in medical physics.

At that time, the profession was still disorganized, even embryonic. Few medical physicists bothered to sit for the American Board of Radiology exam. There was no agreement as to who was qualified to provide clinical physics services. There were no official clinical training programs; there were only a few academic programs, post‐doctoral positions, and a smattering of short training courses. Moving from these early structures to the disciplined, complex, and rigorous education, research science and professional culture we have today has been quite an adventure for all of us. We have known instinctively that what we did was right, but where has it led us?

The developments in radiation imaging, therapy, and technology during my professional life are astounding. Surely, these developments would bring greater dignity, job satisfaction, alleviation of stress, higher salary, and job security, right? Surely applied medical technology will raise the standard for the human condition? Surely, it was both inevitable and cost‐effective? Today we purchase many more quality‐adjusted‐life‐years than ever before and at a lower cost, don't we? Regrettably, the answer is a little checkered.

Along with the complexity of the imaging processes and therapy dose distributions comes the difficulty in interpretation of the medical images, and the difficulty of validating the complex radiotherapy treatment. How long will it be before Artificial Intelligence (AI) does a better job at interpreting an image than a human? How long will it be before automated processes and AI do a better job validating treatments than a human? What then is the role of the human? Is it just to assume the responsibility for the process and to take the blame when the system fails? Does this really serve the better interest of the patient and/or save money? And with all this new technology, are our training programs meeting the standard? Are we keeping up? There is reason to believe we cannot afford to be complacent about our culture and our accomplishments.

Consider these somewhat random thoughts:

“The ability to deal with people is as purchasable a commodity as sugar or coffee and I will pay more for that ability than for any other under the sun.” John D. Rockefeller (https://www.brainyquote.com/quotes/john_d_rockefeller_147467)

“Your company is not a family.” Reid Hoffman, Ben Casnocha, and Chris Yeh (https://hbr.org/2014/06/your-company-is-not-a-family)

“It is difficult to get a man to understand something, when his salary depends on his not understanding it.” Also, “They use everything about the hog except the squeal.” Upton Sinclair (https://www.goodreads.com/author/quotes/23510.Upton_Sinclair) (Yes, I am thinking the professional medical physicist could be the “hog.”)

In 1942, at age 29, David Packard attended a Stanford conference on wartime production. “Somehow, we got into a discussion of the responsibility of management. Professor Holden made the point that management's responsibility is to the shareholders — that's the end of it. And I objected. I said, ‘I think you're absolutely wrong. Management has a responsibility to its employees, it has a responsibility to its customers, it has a responsibility to the community at large’. And they almost laughed me out of the room.” David Packard (https://www.hpalumni.org/hp_way.htm)

If you think you are fortunate to have your job, it could be because your employer agrees with you. If you were to read the minds of many of those administratively in charge of high‐tech medical programs and personnel, you might be surprised at the consensus: “We do not care about you so much; you are lucky to be here and making the salary you enjoy. You think you have a career, but really, you are just a commodity to be bought and sold. And, so are we. We have little or no obligation to provide job security. We will offer as little as we can get by with respect to education, training, or career development for you and your family. We will pay you the lowest amount we can negotiate, and we are much better at that than you are. We would like to make you a contract employee so that we do not have to provide benefits such as health care or a retirement plan. Diversity is a legal matter only; and we pay it lip service and comply. We expect your job to be stressful, and we expect you to develop the personal resources to cope with it. You should not expect to have privacy here; we will read your email. If older employees expect to be paid more, they should be willing to share ever higher burdens of productivity and stress. If they burn out, they may be replaced by someone much younger and much less expensive; that is OK with us. You probably signed an at‐will employment contract, meaning you can be fired at any time for any reason or for no reason at all.”

Are things really that antagonistic in medical physics? No, not yet; but they could be moving in that direction. Right now, employment in a university offers a little protection for some employees, but it may come at a cost in other areas. Some universities have not provided a raise or cost‐of‐living adjustment to workers in years.

Although the technology emanating from Silicon Valley has brought many waves of positive change to our profession, it has not necessarily improved its working conditions or overall job satisfaction without exception. While medical physicist salaries remain solidly high and upper middle‐class, and our job security is (currently) relatively good, this condition is the exception rather than the rule for high‐technology employment. The profession may be at risk if we cannot meet the prevailing scientific, educational, professional and cultural challenges. Thus, the state of the medical physics profession in 2019 is cautiously optimistic, but we must remain vigilant and nimble and be willing to have our professional roles evolve if we are to remain highly‐valued professionals in the imaging and therapy practices of the future.

